# Clinical utility of DaTscan in patients with suspected Parkinsonian syndrome: a systematic review and meta-analysis

**DOI:** 10.1038/s41531-021-00185-8

**Published:** 2021-05-24

**Authors:** Danny Bega, Phillip H. Kuo, Anastasia Chalkidou, Mariusz T. Grzeda, Thomas Macmillan, Christine Brand, Zulfiqar H. Sheikh, Angelo Antonini

**Affiliations:** 1 Northwestern University Feinberg School of Medicine, Chicago, IL USA; 2 Departments of Medical Imaging Medicine, and Biomedical Engineering, University of Arizona, Tucson, AZ USA; 3 King’s Technology Evaluation Centre, School of Biomedical Engineering and Imaging Sciences, King’s College London, London, UK; 4 GE Healthcare, Marlborough, MA USA; 5 GE Healthcare, Pollards Wood, Nightingales Ln, Chalfont Saint Giles, UK; 6 Department of Neuroscience, University of Padua, Padua, Italy

**Keywords:** Parkinson's disease, Diagnostic markers

## Abstract

Images of DaTscan (ioflupane [123I] SPECT) have been used as an adjunct to clinical diagnosis to facilitate the differential diagnosis of neurodegenerative (ND) Parkinsonian Syndrome (PS) vs. non-dopamine deficiency aetiologies of Parkinsonism. Despite several systematic reviews having summarised the evidence on diagnostic accuracy, the impact of imaging results on clinical utility has not been systematically assessed. Our objective was to examine the available evidence on the clinical utility of DaTscan imaging in changing diagnosis and subsequent management of patients with suspected PS. We performed a systematic review of published studies of clinical utility from 2000 to 2019 without language restrictions. A meta-analysis of change in diagnosis and management rates reported from each study was performed using a random-effects model and logit transformation. Sub-group analysis, meta-regression and sensitivity analysis was performed to explore heterogeneity. Twenty studies met the inclusion criteria. Thirteen of these contributed to the meta-analyses including 950 and 779 patients with a reported change in management and change in diagnosis, respectively. The use of DaTscan imaging resulted in a change in management in 54% (95% CI: 47–61%) of patients. Change in diagnosis occurred in 31% (95% CI: 22–42%) of patients. The two pooled analyses were characterised by high levels of heterogeneity. Our systematic review and meta-analysis show that imaging with DaTscan was associated with a change in management in approximately half the patients tested and the diagnosis was modified in one third. Regardless of time from symptom onset to scan results, these changes were consistent. Further research focusing on specific patient subgroups could provide additional evidence on the impact on clinical outcomes.

## Introduction

Dopamine transporter imaging (DaT) has been used in clinical practice to detect dopaminergic deficits in neuro-degenerative (ND) Parkinsonian syndromes (PS) including Parkinson disease (PD) and support the differential diagnosis of non-dopamine deficiency aetiologies (non-DDA) of Parkinsonism in cases of clinical uncertainty^1,2^.

Essential tremor (ET), drug-induced Parkinsonism (DIP) and other forms of non-DDA may present clinical features—bradykinesia, atypical tremor, rigidity, postural instability/gait impairment-resembling PS or PD without evidence of dopaminergic deficit^3^. Therefore, clinically uncertain parkinsonian syndrome (CUPS) presents an important challenge to clinicians because an accurate diagnosis is required in order to provide patients with the appropriate therapies and prognosis. SPECT imaging with DaTscan^TM^ (Ioflupane 123-I SPECT), can provide evidence of pre-synaptic dopaminergic deficits in vivo^4^.

The diagnostic accuracy of DaTscan images has been explored previously^5^ reporting over 90% sensitivity and specificity to differentiate between ND PS and non-DDA diseases. Despite these results, clinicians often rely on clinical judgement and follow-up alone to establish a diagnosis. A number of studies have reported the impact of DaT imaging on clinical utility suggesting that its use in the diagnostic work-up can shorten time to diagnosis and change therapeutic management^6,7^. The purpose of this systematic review and meta-analysis is to examine the available evidence on the clinical utility of DaTscan image results in changing diagnoses and subsequent management of patients.

## Results

### Included studies

The searches retrieved 3916 unique records and 20 studies were included in quantitative synthesis^6–25^. A PRISMA flow diagram is available in Supplementary Fig. 1. Studies’ authors were contacted, where necessary, to confirm or clarify key study details.

Four of the included studies were prospective, including 1 randomised controlled trial (RCT), with the other 16 studies featuring a retrospective design. In total, the studies included 3185 patients. Mean age was 64 years old and ranged from 59^15^ to 78.7 years old^11^. Overall, there was a small majority of male patients (56.4% of the total population). Clinical assessment was based on the UK Parkinson Disease Brain Bank criteria in 5 studies, Hoehn & Yahr stage (H&Y) in 4 studies and Movement Disorder Society-Parkinson Disease criteria (MDS-PD) in 2 studies, the method of clinical assessment was not reported in the rest. The time since onset of symptoms to DaTscan imaging was reported in 11 studies: the means ranged from 2.4 to 6.7 years (median 3.8 years). The method of image analysis used to interpret the SPECT results were explicitly reported in 14 studies with 6 studies reporting visual assessment, 2 quantitative/semi-quantitative, 6 both and missing in the remaining 6 studies. The studies included heterogeneous patient populations, with a variety of indications for performing DaT SPECT. Among the studies, the most common reasons for ordering dopaminergic imaging were to distinguish PD from ET, DIP, early Parkinsonism, vascular Parkinsonism, Lewy Body Dementia (LBD), and dystonia. Details of the studies and patient characteristics are listed in Table 1.

**Table 1:** Overall study characteristics.

Study	Population (*n*)	Country	Design	Mean age (years)	Male (%)	Mean follow-up (months)	Clinical assessment	Image interpretation	Time since onset of symptoms (years)	Diagnosis at baseline/reason for scan
Bairactaris et al.^6^	61	Greece	Prospective, single-arm	64.73 ± 13.60	61%	12	NR	Semi-quantitative	4.36	PD (34), ET (15), VP (3), DIP (1) Other (8)
Bega et al.^7^	83	USA	Retrospective, single-arm	NR	NR	NR	UK PD Brain Bank	Visual and semi-quantitative	NR	PD vs. ET (18), PD vs. DIP (18), PD vs. VP (12), PD vs. normal variant (11), Multifactorial gait D/o (7), Patient requested (4), uncertain PD (3), Unusual tremor (2), Known Dx of MS (2), Myelopathic signs (2), Dystonic features (1), Myoclonus (1), Psychogenic (1), Young-onset with atypical features (1)
Bhattacharjee et al.^10^	256	UK	Retrospective, single-arm	71.65 ± 16.02	53%	NR	modified H&Y/UK PD Brain Bank	Visual and semi-quantitative	4.35	PD (190), VP (5), ET (18), DIP (8), Other (8) LBD (22), Diagnosis Uncertain (5)
Crotty et al.^11^	261	Ireland	Retrospective, single-arm	65.6 ± 12.22	57%	NR	NR	NR	NR	PS (163), LBD (7), DIP (45), PD vs. ET (12), PD vs. dementia (2), PS vs. PD (19), PD progression (2), PD vs. VP (8), PD vs. dystonia (2), unclear (1)
Garcia Vicente et al.^12^	42	Spain	Retrospective, single-arm	78.7 (50–88)	38%	12	NR	Visual and semi-quantitative	NR	PD (19), VP (14), ET (5), DIP (2), Other (2)
Graebner et al.^13^	27	USA	Prospective, single-arm	61.2 ± 13.4	52%	1	NR	NR	4.1	DP vs. ET (5), DP vs. VP (4), DP vs. DIP (4), DP vs. psychogenic or malingering (3), DP vs. others (6), Patient request (4)
Hesse et al.^14^	278	Germany	Retrospective, single-arm	63 ± 12	59%	18	UK PD Brain Bank/MDS-PD/PSP criteria	NR	4.25	PD (129), MSA (10), PSP (8), VP (15), SWEDD (116)
Jennings et al.^15^	35	USA	Retrospective, single-arm	66.8 (46.2–81.7)	63%	6	NR	Quantitative	2.4	PS (30), non-PS (5)
Kupsch et al.^16^	113	Europe/USA	RCT (DaTscan arm)	67.08 ± 11.93	56%	1 and 3	H&Y	Visual	2.45	PS (69), non-PS (26), inconclusive (13)
Løkkegaard et al.^17^	58	Denmark	Retrospective, single-arm	59 ± 14	97%	14	UK PD Brain Bank	Visual	6.76	PD (16), possible PD (41), possible PD plus (19), DIP (4), dystonia (6), ET (4)
Marek et al.^18^	701	USA/Canada	Retrospective, re-analysis of RCT data	60	73%	21	H&Y	NR	NR	NR
Marshall et al.^19^	150	UK	Retrospective, single-arm	63 ± 12	44%	28.8	UK PD Brain Bank	Visual	2.7	Tremor (112), gait abnormality (18), mixed features (20)
Mirpour et al.^20^	134	USA	Retrospective, single-arm	64.4 ± 12.6	78%	17	NR	Visual and semi-quantitative	3	Tremor (59), tone (37), postural ability (46), symptom predominance (46)
Oravivattanakul et al.^21^	175	USA	Retrospective, single-arm	63 (33–88)	53%	NR	NR	Visual	NR	PD (70), DIP/VP/NPH/hypermanganese/structural lesions/stiff-person syndrome/toxic-metabolic/Fragile X–associated ataxia syndrome/psychogenic parkinsonism (46), ET (14), uncertain diagnosis (45)
Sadasivan et al.^22^	65	USA	Retrospective, single-arm	64 (18–88)	49%	7.5	NR	NR	NR	Parkinsonism without tremor (33), Tremor predominant parkinsonism (19), Gait disorder predominant parkinsonism (13)
Siefert et al.^23^	112	USA	Retrospective, single-arm	NR	NR	NR	NR	Visual	NR	Ambiguous (70), not responding to treatment (51), patient considering surgery (2), considered for clinical trial (1)
Sixel-Doring et al.^24^	125	Germany	Retrospective, single-arm	65.5 ± 9.3 (non-Parkinsonian) 60.6 ± 11.7 (possible PD)	54%	NR	MDS-PD	Visual and semi-quantitative	3.84	PD (85), non-Parkinsonian tremor (40)
Thiriez et al.^25^	516	France	Retrospective, single-arm	62.6 ± 12.6	56%	NR	NR	Visual and semi-quantitative	NR	NR
Tolosa et al.^26^	118	8 EU countries	Prospective, single-arm	65.5 ± 11.2	50%	NR	H&Y	Visual	3.8	PD (59), other PS (8), ET (16), other non-pre-synaptic PS (10), inconclusive (25)
Yomtoob et al.^27^	55	USA	Retrospective, single-arm	64.549	55%	0–24	NR	NR	NR	PD vs. DIP

*CD* cerebellar disorder, *DIP* drug-induced parkinsonism, *ET* essential tremor, *H&Y* Hoehn & Yahr stage, *MSA* multiple system atrophy, *NR* not reported, *PD* Parkinson’s Disease, *PS* Parkinsonian syndrome, *PSP* progressive supranuclear palsy, *VP* vascular Parkisonism.

### Data extraction and quality assessment

There was an unclear risk of bias for the Patient selection domain due to poor description of patient recruitment and selection criteria. The Index test was generally well described and there were no major concerns for introducing bias. In the Flow and Timing domain, with the exception of 4 studies, the rest of the studies lacked clarity in the time interval between clinical diagnosis and DaTscan. The majority of the studies did not report intra- and inter-observer variation. All studies included a reference standard (clinical diagnosis) albeit with a variety of methods reported. The only study to report a sample size calculation was the RCT by Kupsch et al.^7^. With the exception of Hesse et al.^13^ and Marshall et al.^17^, it was unclear if structural brain imaging was available for comparison with the ioflupane image results. Table 2 lists the change in management and diagnosis extracted from each study. A detailed breakdown of the risk of bias assessment results is presented in Supplementary Table 1.

**Table 2:** Study outcome data for change in management and diagnosis.

Study	Population (*n*)	Change in management	Change in diagnosis	Drug changes^a^	Other change in management
Bairactaris et al.^6^	61	NR	34%	NR	NR
Bega et al.^7^	83	37%	43%	33%	4%
Bhattacharjee et al.^10^	256	68%	46%	68%	NR
Crotty et al.^11^	81	65%	30%	35%	NR
Garcia Vicente et al.^12^	42	17%	NR	17%	NR
Graebner et al.^13^	27	67%	NR	52%	NR
Hesse et al.^14^	278	NR	55%	NR	NR
Jennings et al.^15^	35	NR	NR	NR	NR
Kupsch et al.^16^	113	49%	40%	50%%	17%
Løkkegaard et al.^17^	58	43%	NR	NR	NR
Marek et al.^18^	701	NR	8%	NR	NR
Marshall et al.^19^	150	NR	49%	21%	NR
Mirpour et al.^20^	134	49%	NR	36%	13%
Oravivattanakul et al.^21^	175	NR	NR	NR	NR
Sadasivan et al.^22^	65	63%	20%	47%	6%
Siefert et al.^23^	112	58%	28%	NR	NR
Sixel-Doring et al.^24^	125	NR	6%	NR	NR
Thiriez et al.^25^	516	60%	28%	NR	NR
Tolosa et al.^26^	118	72%	52%	36%	36%
Yomtoob et al.^27^	55	38%	NR	38%	NR

*NR* not reported.

^a^Drug changes constituted, start a new drug, change in dose or discontinue a drug.

### Change in management and change in diagnosis

**Fig. 1:**
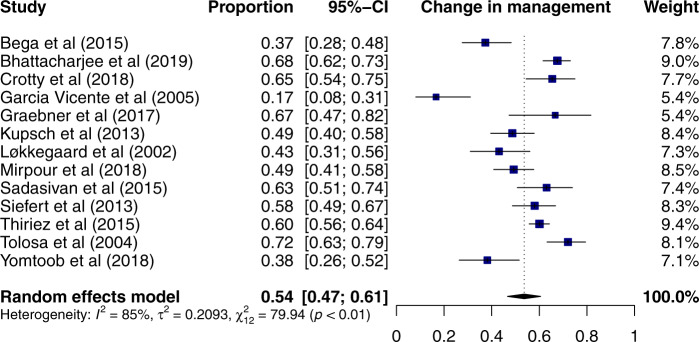
Forest plot of the meta-analysis of studies reporting change in management with DaTscan using a random-effects model. The studies are ordered alphabetically.

**Fig. 2:**
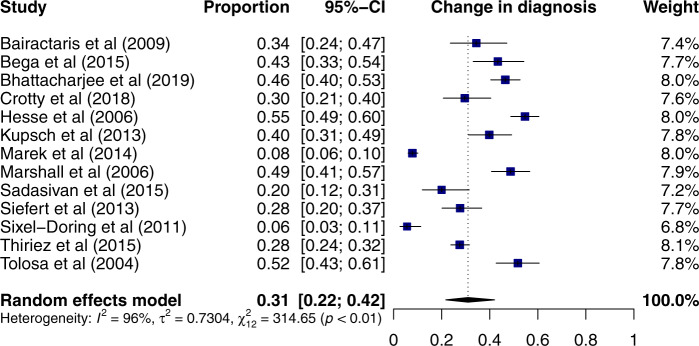
Forest plot of the meta-analysis of studies reporting change in diagnosis with DaTscan using a random-effects model. The studies are ordered alphabetically.

The primary endpoints were the pooled analysis of the proportions of patients who had a change in management and in diagnosis following imaging with DaTscan. Change in management was reported by 13 studies^6,7,9–12,18,20,21,23–25^ including 950 patients. Using a random-effects model 54% (95% CI: 47–61%) of patients had a change in management following the scan (Fig. 1). Change in diagnosis was reported by 13 studies^6–10,13,16,17,20–24^ including 779 patients. Using a random-effects model, DaT imaging resulted in a change of diagnosis in 31% (95% CI: 22–42%) of patients (Fig. 2). With the exception of one study^6^, change in management was higher than a change in diagnosis. The two pooled analyses are characterised by high levels of heterogeneity (*I*^2^ of 85% and 96% for change in management and change in diagnosis, respectively, both *p* < 0.01). Further sub-group analyses were performed to explore the high levels of heterogeneity.

### Sub-group analyses

Based on the pre-existing diagnosis, change in management and diagnosis varied between 33% and 70% and 35%–56%, respectively. Variability in the definition of clinically uncertain patients in the included studies prohibited further sub-group analysis for this cohort. Further exploratory subgroup analyses were performed based on the region where the study was performed (0.51, 95% CI: 0.41–0.62 North America vs. 0.57, 95% CI: 0.47–0.66 Europe), study design (0.62, 95% CI: 0.49–0.74 prospective vs. 0.54, 95% CI: 0.47–0.62 retrospective), patient age (0.56, 95% CI: 0.42–0.69 < 64 years vs. 0.58, 95% CI: 0.50–0.67 > 64 years), female to male ratio (0.55, 95% CI: 0.48–0.62 < 1 vs. 0.68, 95% CI: 0.55–0.79 > 1), follow-up time (0.54, 95% CI: 0.44–0.64 < 16 months vs. 0.45, 95% CI: 0.32–0.57 > 16 months) and time since onset of symptoms (0.57, 95% CI: 0.41–0.71 < 3.84 years vs. 0.59, 95% CI: 0.43–0.74 > 3.84 years). Additional Forest plots are provided in the Supplementary Figs. 3–16.

### Meta-analysis outcomes—secondary endpoints

The proportion of patients who started a new drug treatment following the scan results, reported in 10 studies, was 26% (95% CI: 21–32%), who had their drug treatment stopped (12 studies) was 14% (95% CI: 10–20%), and who had a change in dose (3 studies) was 6% (95% CI: 2–19%). There was significant heterogeneity for each outcome (*p* < 0.01 for all).

### Sensitivity analysis

We performed a leave-one-out analysis to test the influence of each individual study on the overall pooled change in management and diagnosis rates. Using a random-effects model, all 13 studies were sequentially excluded. Apart from Garcia et al.^11^, none of the other studies had any significant effect on the change in management results (Supplementary Fig. 17). Removing Garcia et al.^11^ from the analysis resulted in a small increase in the change in management. Similarly, for the change in diagnosis, with the exception of Marek et al.^16^, none of the other studies had any significant effect on the change in diagnosis (Supplementary Fig. 18). Removing Marek et al.^16^ from the analysis resulted in a small increase in the change in diagnosis.

### Meta-regression

Studies including patients with higher mean age and higher female to male ratio resulted in a larger change in diagnosis. The latter was also a factor contributing to higher change in management. No other variables were associated with a change in management or change in diagnosis (Supplementary Figs. 19–21). Due to the limited number of studies, only univariate meta-regression was performed. The funnel plot analysis showed distribution asymmetry potentially indicating the presence of publication bias (Supplementary Fig. 22).

## Discussion

This systematic review summarises the existing evidence on the clinical utility of imaging with DaTscan in the diagnosis and management of patients with suspected PS. Our meta-analysis showed that the use of DaT imaging changed management in approximately half the patients tested and altered the diagnosis in one third, even in individuals with long symptom duration.

Currently, the threshold for the proportion of cases that constitute a ‘clinically important’ change in management varies depending on multiple factors, including but not limited to, underlying conditions and available treatment management options. For example, a study analysing the results from the National Oncologic PET Registry proposed a minimum threshold of 25% change based on reported rates from individual studies ranging between 10% and 40%^26^. The authors of the IDEAS study, meanwhile, identified a 30% threshold as being consistent with previous studies of coverage, with evidence examining the association between diagnostic imaging and changes in management^27^. This threshold was also supported by previous work assessing the clinical utility of amyloid PET^28^. In our analysis, with the exception of one outlier study^11^, the change in management ranged from 37% to 72% (mean = 54%), demonstrating a clinically important impact from results of imaging with DaTscan in the management of PD and non-DDA.

It was difficult to quantify the effect of change in management on clinical outcomes as most studies did not report this outcome. Change in management can impact patients’ outcomes in a number of ways including affecting their quality of life by avoiding unnecessary medications and their side-effects and by providing people with clearer expectations related to prognosis. The RCT by Kupsch et al.^7^ showed significantly more changes in management and diagnosis in the group undergoing DaT SPECT compared to the clinical assessment control group, however, quality of life was not significantly different. The study, however, was not powered to detect a difference in the quality of life. In neurogenerative disorders, it is often difficult to capture the long-term effect of diagnostic tests in clinical outcomes. Many of the quality of life measures or other patient-reported outcomes used in these studies are validated for patients with PD and may not be appropriate to assess non-dopamine deficiency aetiologies of Parkinsonism.

The study reporting the lowest change in management included elderly patients with suspected vascular parkinsonism and featuring the highest mean age (78 years old) among all studies^11^. Elderly patients frequently suffer from concomitant cognitive deficits and the presence of cerebrovascular brain pathology potentially limits the benefit from levodopa treatment regardless of the imaging results. These patients may have already received medications without benefit, and this led to the decision to obtain DaT images to support diagnosis and discuss prognosis, rather than to influence management. Previously Antonini et al.^29^ reported that DaTscan imaging helps to manage patients with cerebral vasculopathy and that normal uptake was associated with no benefit from medications in over 90% of participants. A recent systematic review and meta-analysis showed that patients with vascular parkinsonism have indeed a low response rate to levodopa^30^. The combination of an elderly population with potentially cognitive impairment and no benefit from levodopa reduce treatment options in these patients and therefore, determine an overall low rate of change in management. It has also been shown that the rate of change in management can differ between movement disorder specialists and general neurologists^7^.

One of the findings of this systematic review is the variable duration of symptoms, ranging from 2 to 6 years, prior to imaging with DaTscan. The mean time of symptom duration in a systematic review and meta-analysis of diagnostic accuracy studies including patients with unclear Parkinsonism^5^ varied between 2 and 5.6 years with the longest duration observed in patients with a final diagnosis of DIP. This is in contrast to the duration of symptoms reported in early studies testing the effect of levodopa in patients with PD, where the average symptom duration was ~6 months^31^. A possible explanation for the increased symptom duration observed in this study is the prioritisation of a levodopa trial over obtaining further diagnostic imaging tests. Alternatively, the ‘watch and see’ approach may have resulted in prolonged clinical observation prior to obtaining a scan.

It is assumed that in most cases, a longer symptom duration would lead to a clearer clinical presentation of PD^32,33^. In these cases, DaT SPECT will be used to confirm the diagnosis and result in less change in management. The analysis of longitudinal data on diagnostic confidence obtained from the National Parkinson’s Foundation Quality Improvement Initiative reported that diagnostic certainty increases with time. The authors also reported that shorter disease duration, absence of tremor or motor fluctuation, poor cognition, comorbidities, and absence of levodopa treatment were associated with lower diagnostic confidence at baseline^34^. For cases with either very slow or rapid progression, image results with DaTscan may lead to a change in diagnosis and management. The rate of disease progression in clinical studies varies due to patient selection in treatment trials, medication use, and duration of follow‐up. It has been also reported that the median annual progression rates of motor impairment and disability, are subject to considerable individual variability^33^.

This systematic review did not restrict inclusion based on study design. With the exception of two RCTs^7,16^ the rest of the studies were reporting single centre real-world experiences utilising both retrospective and prospective designs. Unsurprisingly, the pooled analysis for change in management and change in diagnosis showed high heterogeneity. Sub-group analysis according to pre-existing diagnosis reduced heterogeneity. This was mostly observed for the change in management outcome. This finding could be due to the different treatment management options for different subgroups. For example, in the differential diagnosis of PD vs. DIP change in management is primarily related to drug changes and may occur in higher frequency than other subgroups. Similarly, for ET vs. PD, a relatively high change in management is to be expected as the two groups are treated differently. With the exception of one study^6^, the rate of change in management was higher than a change in diagnosis. This is to be expected as often DaT SPECT is ordered to confirm a diagnosis but the change in management comes in response to gaining confidence in the original diagnosis. In addition, the definition of ‘change in diagnosis’^6^ also included cases where the pre-scan diagnoses were less definitive and firmly established post-scan. Therefore, in many cases, DaTscan results may improve diagnostic confidence rather than suggest a different medical condition. Most included studies, however, did not report diagnostic confidence prior to imaging limiting the ability to draw robust conclusions on the degree of diagnostic uncertainty. The three studies that reported diagnostic confidence results^12,21,24^, all indicated that post-imaging confidence in the prediction of degenerative parkinsonism decreased in cases of a normal scan but remained high or increased when results were abnormal suggesting that DaT imaging was mainly utilised to confirm the presence of PD.

Heterogeneity remained high after performing sub-group analysis, suggesting that additional sources of heterogeneity are present. Some of the variables considered important for clinical utility, such as diagnostic uncertainty, were inconsistently reported in the primary studies, limiting a comprehensive evaluation of their impact on heterogeneity. In addition, analyses of sources of heterogeneity, and the interpretation of those analyses were complicated by the relatively small sample size that did not allow us to perform multivariable meta-regression to evaluate potential confounding or interaction between variables. The results of the meta-analyses presented here must be interpreted with caution due to the significant heterogeneity in the investigated population.

Our study has few limitations. Firstly, the number of recruited patients in some of the studies is relatively small. However, the absence of publication bias and the analysis of 20 different studies with a total of 3185 patients strengthen our results. The heterogeneity of the studies included in our meta-analysis could be considered as a limitation. We attempted to account for this variation by using random-effects modelling, sub-group, and leave-one-out sensitivity analysis. In addition, study design varied between studies contributing to a high degree of heterogeneity for the pooled effect. The assessment of the methodological quality revealed potential sources of bias in the reviewed studies. In most studies, the time between imaging with DaTscan and clinical assessment was not clearly defined highlighting the fact that this is as much an outcome of inadequate reporting as of trial design. Likewise, the study recruitment period and selection criteria were not adequately described. The above reflects the limitations of retrospective data collection and analysis. However, our subgroup analysis based on study type did not show any influence on the results (0.62, 95% CI: 0.49–0.74 vs. 0.54, 95% CI: 0.47–0.62). This result provides more confidence to our analyses as retrospective studies have a higher propensity for bias, missing data and a tendency to overestimate the effect. Study design heterogeneity also limited a more detailed assessment of the relationship between clinical utility and clinically uncertain diagnosis. Our results were robust to sensitivity analysis.

## Conclusion

In conclusion, our systematic review and meta-analysis showed that the results of imaging with DaTscan were associated with a change in management in approximately half the patients tested and altered the diagnosis in one third even in patients with longer symptom duration. Regardless of time from symptom onset to scan results, these changes were consistent and suggest that earlier execution of DaTscan may shorten time to diagnosis in uncertain cases. Further research focusing on individual patient subgroups could provide evidence on the impact on clinical outcomes.

## Methods

### Search methods

A search of studies published between January 2000 and June 2019 in MEDLINE (Ovid interface), EMBASE (Ovid interface), PsycINFO and the Cochrane Library (CDSR and CENTRAL) was performed. The search was performed with a combination of terms related to the population, DaTscan, and outcomes. Previous reviews on the subject and references reported in the identified studies were also used. The list of titles and abstracts was screened by two independent reviewers (AC and TM) for eligible studies using EndNote X7.8. When results on the same dataset were reported in several publications, only the most recent or complete publication was included in the analysis, in order to avoid overlapping cohorts. Authors were contacted for clarifications when required. The full electronic search strategies are listed in Supplementary Table 1. This systematic review was registered on PROSPERO under the reference CRD42020161315.

### Study selection

To classify for inclusion, studies had to:

- Report the clinical utility analysis of imaging with DaTscan.
- Used clinical examination with or without structural imaging to establish the pre-SPECT diagnosis.
- Have been published in peer-reviewed scientific journals.
- Used either visual or quantitative methods to interpret the DaT image results.
- There were no restrictions for inclusion based on the clinical setting.

The following studies were excluded:

- Studies that combined DaT SPECT with other imaging tests to establish the diagnosis.
- Case studies and small case series (<10 patients).

### Data extraction and quality assessment

Data were extracted by two reviewers (TM and AC) independently and were recorded in absolute numbers. Where proportions only were reported the absolute figure was recalculated. Data on clinical utility following imaging with DaTscan, scan technical characteristics (handling of DaT interfering medication, patient preparation, test interpretation, technical failures, and assessors (e.g. knowledge of other test results)), and baseline characteristics of studied patients were extracted (disease duration, age at imaging, criteria used for diagnosing PD). Change in diagnosis was defined as any change in the patient’s assigned or suspected diagnosis following disclosure of the results of the imaging scan. Change in management was defined as any changes that the study authors recorded as changes in treatment or further investigations, such as drug changes (start of a new drug, discontinue existing medication or change in dose), referral for counselling or physical therapy following the disclosure of DaT imaging results. Results were compared, and discrepancies between the two reviewers were resolved in a meeting.

Risk of bias assessment was carried out on all studies using an adapted form of the QUADAS-2 checklist amended by the study authors to fit studies of clinical utility, rather than diagnostic accuracy. A copy of the amended QUADAS-2 checklist is provided in Supplementary data (Sect. 1.3). The risk of bias assessment was used independently of the quantitative meta-analysis.

### Quantitative analysis

Change in management and diagnosis was pooled with a random-effects model to account for clinical and study design heterogeneity. Sampling variability (standard errors) of all proportions was assessed and their 95% confidence intervals were reported. All proportions were subject to logit transformation to facilitate data analyses under the assumption of normal distribution. The logit transformations and their 95% confidence intervals were converted back to proportions to provide an estimate of the pooled change in management and diagnosis and their heterogeneity. A random-effect model (with restricted maximum-likelihood estimation) was used as it allows for more realistic assumptions of the true between studies heterogeneity^35^. The between-study variance was estimated with the DerSimonian and Laird method^36^. Summary effect sizes were estimated as weighted means of observed effect sizes of individual studies. The level of heterogeneity was assessed using the between-study variance (τ-squared statistic), *Q* statistics, and *I*-squared statistics. All results were summarised in forest plots. Any observed heterogeneity was explored using pre-specified sub-group and meta-regression analysis. Sensitivity analysis was also performed to test the validity and stability of the results. All the analyses were performed in the R programme (v 3.6.1) using ‘metafor’ and ‘meta’.

### Sub-group analyses

The results of meta-analyses for change in management and diagnosis were subjected to sub-group analysis by regional variation, the proportion of patients with diagnoses deemed clinically uncertain and time since onset of symptoms (a categorical variable was created for each sub-group). Additionally, separate meta-analyses were carried out by pre-scan diagnosis in studies that reported these separately (PD vs. ET, PD vs. DIP, early-onset PD, PD vs. vascular dementia, PD vs. dystonia).

### Meta-regression

The following predictors were checked in meta-regression analysis: year of publication (continuous), a number of patients (continuous), study region (categorical: Europe vs North America); study design (categorical: a retrospective, single-arm vs other design); mean age (continuous); females to males ratio (continuous), follow-up time (continuous; months); clinical assessment (UK PD Brain Bank/MDS); image interpretation (categorical: quantitative vs visual vs both), time since onset of symptoms (continuous: in years).

## Supplementary information

Supplemental material

## Data Availability

All data included in this manuscript have been sourced by publicly available information and are provided in the main manuscript and the supplementary data.
