# Supplementary material for: Clinical utility of DaTscan in patients with suspected Parkinsonian syndrome: a systematic review and meta-analysis
Source: NPJ Parkinsons Dis. 2021 May 24;7:43. doi: 10.1038/s41531-021-00185-8 (PMC8144619; doi:10.1038/s41531-021-00185-8)
Supplement: Supplementary file 1 — Supplemental material [file 41531_2021_185_MOESM1_ESM.pdf]

Supplementary data

Search strategies

Total references retrieved = 5877

Total following de-duplication = 3916

Supplementary Table 1: Example of the search strategy used for Ovid MEDLINE(R) and Epub Ahead of Print, In-Process & Other Non-Indexed Citations and Daily 1946 to June 04, 2019, search date: 4th June 2019

1	exp Parkinsonian Disorders/	75016
2	((parkinson* adj2 (symptom* or disease* or syndrom* or degenerat* or disorder*)) or (lewy adj3 bod*)).tw.	95434
3	(motor adj3 (disorder* or disease* or d?sfunction*)).tw.	19455
4	(movement* adj3 (disorder* or disease* or d?sfunction*)).tw.	16820
5	((uncertain or equivoc* or susp* or indicat*) adj3 parkin*).tw.	608
6	(dopamine* adj3 (degenerat* or d?sfunction*)).tw.	4456
7	or/1-6	139997
8	Dopamine Plasma Membrane Transport Proteins/ or dopamine transport*.af.	8178
9	(DaT adj3 imag*).af.	234
10	(Dat?scan* or dat scan* or dat?spect* or dat spect*).af.	444
11	Ioflupane*.af.	226
12	("123ip" or "123?ip" or "123 ip" or 123i-FP-CIT or FPCIT or FP-CIT or beta?CIT or beta CIT or CIT?SPECT or CIT SPECT).af.	1211
13	or/8-12	8870
14	(SPECT or SPET or SPET-CT).tw.	28168
15	exp Tomography, Emission-Computed, Single-Photon/ or exp Radionuclide Imaging/	204725
16	(dopamine transport* or striatal dopamine*).tw. or *Dopamine/	46173
17	(14 or 15) and 16	3138
18	13 or 17	10232
19	7 and 18	3428
20	exp "sensitivity and specificity"/	553583
21	(sensitivit* or specificit* or accurac* or gold standard* or reference standard*).tw.	1340117

22	((predictive adj3 value$) or (roc adj curve$) or AUC or area under the curve).tw.	197728
23	((false adj positiv$) or false negativ$).tw.	73096
24	((observer adj variation$) or (likelihood adj3 ratio$)).tw.	15721
25	likelihood function/	21195
26	exp mass screening/	121182
27	exp Diagnostic errors/ or ((diag* or dx) adj2 chang*).tw.	117939
28	(diagnos* adj2 certain*).tw.	3689
29	differential diagnos*.tw.	122201
30	prescan diagnos*.tw.	5
31	(diagnos* adj3 Parkinson*).tw.	2543
32	(di or du).fs.	2449342
33	or/20-32	4064088
34	19 and 33	1025
35	animals/ not (animals/ and humans/)	4553169
36	34 not 35	952
**37**	**limit 36 to yr="2000 -Current"**	**909**

PRISMA flowchart

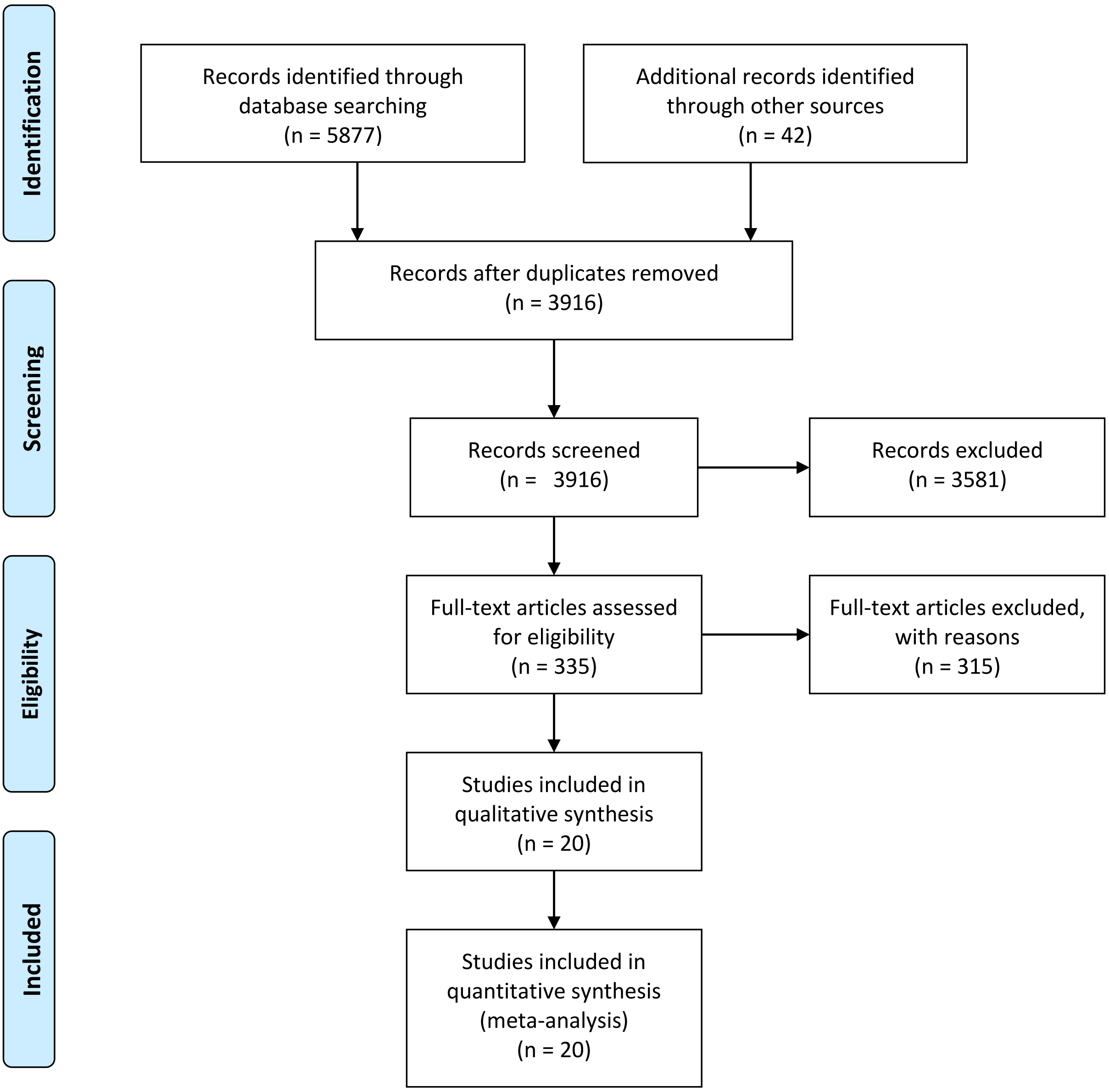

Supplementary Figure 1

Methodological quality assessment

Domain 1: Patient selection

A. Risk of bias		Answer
Q1A Was a consecutive or random sample of patients enrolled?	As stated	
Q1B Was a case-control design avoided?	Studies that included patients with uncertain diagnosis score as ‘yes’. Studies that included healthy volunteers/patients confirmed as PD negative score as ‘no’. If no information is provided score as ‘unclear’	
Q1C Did the study avoid inappropriate exclusions?	If the study has excluded ‘difficult to diagnose’ patients score this as ‘no’. If no information is provided score as ‘unclear’.	
Q1D Was the sample size appropriate?	Was there a sample size calculation performed? If ‘yes’ then please provide a comment whether or not this was adequate. If clinical utility is a secondary outcome then by definition this will be scored as ‘no’	
Q1E Could the selection of patients have introduced bias?	If a patients were considered eligible for the study when the clinical data posed significant uncertainty to the neurologist to establish a clinical diagnosis of parkinsonism and the study listed or referenced criteria of uncertainty such as ‘’*criteria were assessed by referring neurologists and included at least one of the following: only one of the three cardinal signs of parkinsonism, with or without asymmetry; two signs without bradykinesia; atypical signs; signs of mild intensity; poor response to L -DOPA, and lack of disease progression (Kupsch, Bajaj et al. 2013).*’’ then score this item as no. If no information is provided then score this item as ‘unclear’ for example *‘We reviewed the files of all consecutive patients who underwent a [123I]-FP-CIT SPECT examination in the nuclear medicine department (Thiriez, Itti et al. 2015).’*	
B. Concerns regarding applicability		
Q1F Is there concern that the included patients do not match the review question?	Please state any concerns you may have for the included patient population.	

Domain 2: Index test

A. Risk of bias		Answer
Q2A Were the index test results interpreted without knowledge of the results of the reference standard?	-If the DaTSCAN was carried out and reported by neuroimaging expert nuclear medicine physicians that were blinded to the patients' clinical diagnosis then score this as 'yes'. If no information is provided score as 'unclear'.<br>-If the post-DaTSCAN diagnosis is formulated by a consensus panel or clinician blinded to the pre-DaTSCAN diagnosis, then score as 'yes'. If the same clinician provided the pre- and post-DaTSCAN diagnosis, then score as 'no'. If no information is provided score as 'unclear'.	
Q2B If a threshold was used, was it pre-specified?	If the DaTSCAN result was interpreted based on predefined criteria listed by the authors then score this item as 'yes'. For example *'Images were also analyzed semi-quantitatively with semiautomatic method using template regions of interest, as described in earlier publications* (two references provided)' or *'As per the visual assessment method, grade 1 abnormality appears like a "full-stop with a disappearing comma" (asymmetrical loss ofputaminal tail), grade 2 shows "two full-stops" (bilateral loss of putaminal tail), and grade 3 shows "disappearing full stops" (partial to complete loss of caudate and putaminal signal)'.* If no information is provided score as 'unclear'.	
Q2C Could the conduct or interpretation of the index test have introduced bias?	Could the visual or quantitative interpretation of DaTSCAN introduce bias? Examples of potential sources of bias are as follows: Scanner model, injected dose, uptake period, emission time, attenuation correction method, reconstruction method, volume of interest delineation method. Measures to restrict motion or motion correction method. Also withdrawal of dopaminergic medication at the time of SPECT examination?	
B. Concerns regarding applicability		
Q2D Is there concern that the index test, its conduct, or interpretation differ from the review question?	Is there any aspect of the DaTSCAN conduct or interpretation that may have introduced bias?	

Domain 3: Reference standard

A. Risk of Bias		Answer
Q3A Is the reference standard likely to correctly classify the target condition?	The reference standard might be different for different conditions. For the diagnosis of PD for example, if the study used internationally recognised diagnostic criteria such as the United Kingdom Parkinson's Disease Brain Bank's criteria for idiopathic PD, then score this item as 'yes'. If no information is provided score as 'unclear'.	
Q3B Were the reference standard results interpreted without knowledge of the results of the index test?	If the reference standard result used to form a pre-DaTSCAN diagnosis was interpreted without knowledge of the DaTSCAN please score this item as 'yes'. If no information is provided score as 'unclear'.	
Q3C Could the reference standard, its conduct, or its interpretation have introduced bias?	As stated.	
B. Concerns regarding applicability		
Q3D Is there concern that the target condition as defined by the reference standard does not match the review question?	Please state any concerns you may have for the choice of reference standard.	

Domain 4: Flow and timing

A. Risk of bias		Answer
Q4A Was there an appropriate interval between index test(s) and reference standard?	Please note this item has two components listed as separate bullet points below:<br>-If the original diagnosis and the diagnosis based on DaTSCAN were established within a 12 weeks period then score this item as 'yes'.<br>- If the study follow-up to confirm the diagnosis was at least 2 years score this item as 'yes'.<br>This item is scored 'unclear' when no information is given or when the time of either the reference or the index test is missing.	
Q4B Did all patients receive a reference standard?	If it is clear from the study that all participants (or a random selection) who received a DaTSCAN also received a diagnostic test pre-DaTSCAN, then this item should be scored as 'yes.' If some of the participants who received	

	a DaTSCAN did not receive a pre-DaTSCAN diagnosis (or the selection was not random), then this item should be scored as 'no.' If this information is not reported, this item should be scored as 'unclear.'	
Q4C Did patients receive the same reference standard?	If it is clear from the study that all participants received a DaTSCAN also received the **same** pre-DaTSCAN diagnostic testing, then this item should be scored as 'yes.' If not all of the participants who received the same pre-DaTSCAN diagnostic testing, then this item should be scored as 'no.' If this information is not reported, this item should be scored as 'unclear.'	
Q4D Were all patients included in the analysis?	If not, was an explanation provided? Is the explanation sufficient to exclude bias?	
Q4E Could the patient flow have introduced bias?	Is there any aspect of the study design timing, including timing of the DaTSCAN or the reference test that could have introduced bias?	

Additional items for assessing methodological quality in index test domain (McCleery et al, 2015).

Question	Judgement	Criteria
1: Were uninterpretable or intermediate test results reported?	Yes	The number or proportion of uninterpretable or intermediate test results is reported.
	No	Uninterpretable or intermediate test results arose but the number or proportion is not reported.
	Unclear	It is not possible to tell whether there were any uninterpretable or intermediate test results.
2: Were structural brain images available for comparison?	Yes	Structural brain images taken within 6 months of the DaTSCAN images were available to aid interpretation.
	No	No structural brain images (± 6 months) were available to aid image interpretation.
	Unclear	Insufficient information to make a judgement.
3: Was the method of image reconstruction consistent throughout the study?	Yes	The method of image reconstruction is stated and was the same for all participants in the study.
	No	The method of image reconstruction varied within the study.
	Unclear	Insufficient information to make a judgement.
4: Had test operators had appropriate training?	Yes	DaTSCAN image interpreters were fully qualified or certified nuclear medicine

		specialists with prior experience of the technique.
	No	DaTSCAN image interpreters lacked this training or experience.
	Unclear	Insufficient information to make a judgement.
5: Were data on observer variation in DaTSCAN image interpretation reported and within an acceptable range?	Yes	Data on intra- and inter-observer variation in DaTSCAN image interpretation are reported and agreement is good (kappa > 0.6).
	No	Observer variation is not reported or agreement was poor (kappa < 0.6).
	Unclear	It is not clear whether observer variation was measured.

Meta-analyses methods

Quantitative results of this systematic review were subject of meta-analysis in which outcomes from all primary studies were synthesized (Borenstein, Hedges, Higgins, & Rothstein, 2009; Schwarzer, Carpenter, & Rücker, 2015).

Logit transformation of proportions

All outcome measures included in this meta-analysis were reported as proportions in primary studies. To perform the analyses they had to be transformed to logit units (Wang, 2018). Logit transformation of proportions was required to satisfy the assumption of normal distribution, which is the key assumption on which statistical models and tests of significance used in this study are relying. Logit transformation of proportions is often used when dealing with proportional data, although other options like double arc-sine transformation or conducting analyses on untransformed data are also used (Wang, 2018). For this meta-analysis all available options were considered carefully. The option of no transformation was ruled out first as it requires a symmetrical distribution of proportions and all effect sizes to have values between the limits of 0.2 and 0.8. Otherwise it may potentially lead to biased estimates and misleading results of statistical inference testing. The other alternative considered, was the arc-sine option. This option performs best when the sample sizes are small and there are outliers in the data than needs to be handled (Viechtbauer, 2010; Wang, 2018). The latter was not the case in the current study and it was decided to undertake the simplest option of logit transformation. For a more detailed discussion on the above options please see (Wang, 2018).

Once all the analyses have been finished, for reporting purposes, all the estimates in logits and their associated confidence intervals were converted back to original proportions to simplify the interpretation of the results and make them more meaningful to readers. Summaries of study effects for each reported meta-analysis outcome was illustrated with forest plots.

Random effects model

There are two main options for calculating the meta-analyses effect sizes either the fixed or the random effects model. The fixed effect model provides the framework for studies based on the assumption that all studies share a common effect, and all observed differences are attributed to a random error. In other words, it is assumed that the true effect sizes are identical across all studies and vary only due to sampling differences. In the fixed effect model, all studies are weighted by the inverse of their sampling variances which is a function of proportions and sample sizes of studies included in the analysis (Borenstein et al., 2009; Wang, 2018).

In the case of a random effects model studies are allowed to differ on their effect sizes and additionally the sampling error within studies is taken into account. The model assumes that the total observed variance of effect sizes can be attributed to two parameters: the between-study variance, which is the part representing 'true' variability responsible for the real or systematic differences among studies, and the within-study variance being the effect of sampling variability of studies. In meta-analysis the between study variance is regarded as one of the key indicators of study effects heterogeneity. It is denoted by $\boldsymbol{\tau}^2$ and various methods exist to estimate its value. The most populars are the maximum likelihood (Hardy & Thompson, 1996), restricted maximum likelihood estimation (Raudenbush & Bryk, 1985, 2002) and DerSimonian Laird method based on moments (DerSimonian & Laird, 1986; Wang, 2018). All of them have their merits and perils but their results are usually rather similar and rarely lead to different conclusions.

Weights of the studies in this approach are based on the inverse of variance of studies. It is worth noting, however, that in the random effects model this variance is the sum of the above mentioned components: the within- and between-study variances.

In the current analyses it was decided to use a random effects model as it allows more realistic assumptions of the possible true heterogeneity of effects across studies (Borenstein et al., 2009). The between study variance was estimated with DerSimonian and Laird methods as recommended by Wang (2018). Summary effect sizes were estimated as weighted means of observed effect sizes of individual studies.

Outlying and influential studies detection

The influence of individual studies on the results was tested using 'leave one out' sensitivity analysis. This method allows to iteratively exclude each study from the analysis and to recalculate the size of the effect using only the remaining studies. Another approach was based on the analysis of residuals (z-values).

Heterogeneity quantified

Several indices to measure heterogeneity in meta-analyses exist. First measure of heterogeneity used here was the level of between study variance represented by τ^2. This statistic has already been introduced above. Since τ^2 is the measure of absolute units its interpretation is rather difficult without additional tests. One of such tests is Q statistic. It is a formal test of the null hypothesis stating that $\tau^2 = 0$ which amounts to the testing the hypothesis whether there is significant level of heterogeneity of effect sizes. The p-values for this test are reported on forest plots. Finally, in the current analyses heterogeneity was also measured using I^2. It represents the ratio of between study variance to total variance of studies (Borenstein et al., 2009). It was assumed that high heterogeneity is defined by values of I^2 greater than 50% (Higgins & Thompson, 2002). The above indices, their 95% confidence intervals (τ^2 , I^2) and p values (Q statistics) were reviewed and their summary information was used to interpreter the level of heterogeneity in the reported summary effects.

Moderator analysis (or searching for factors explaining heterogeneity)

For the domains with confirmed substantial level of heterogeneity further procedures were employed aiming at searching for possible explanation of observed differences in effect sizes between studies. A subgroup analysis and meta-regression analyses were used for this purpose.

Subgroup analyses

For the subgroup analyses, we used categorical variables from study level characteristics, usually with no more than two categories. When the original variable was continuous it was categorized according to the median. The summary effect sizes for each category were estimated using the random effects model as well. The between study variances of effects from given subgroups were pooled using estimations of overall summary effects. All summaries of study effects for each domain and given subgroup were illustrated with forest plots.

Meta-regression models

Meta-regression was another approach in the process of exploration of possible factors underlying observed level of heterogeneity of study effect sizes. Unfortunately due to the limited number of studies it was not possible to construct multivariable models with more than one predictor. We followed the known rule thumb that there should be at least 10 studies per predictor in meta-regression analysis and performed separate univariate meta-regression models instead. Their effects were reported as beta coefficients and R^2 indexes. Relations between outcomes and all tested

moderator variables were graphically illustrated with scatter plots. All models were estimated under random effects model (Borenstein et al., 2009).

Publication bias

The final point of statistical analyses was the issue of publication bias. Although it is still under discussion in the literature whether it makes sense to include this part of meta-analysis for proportions as primary outcomes in which most them are reported from observational studies we decided to include the relevant output for this issue. In the current study, we present results of this type of analysis in a graphical form of the funnel plots followed by the formal test of the hypothesis that funnel plot is symmetrical (Wang, 2018).

Software information

All the analyses were performed in R programme (v 3.6.1). Two dedicated packages were used in for analyses and graphics which were *metaphor* (Viechtbauer, 2010) and *meta* (Schwarzer, 2010).

Methodological quality assessment

	Patient selection						Index test				Reference standard				Flow and timing					Additional items				
STUDY	Q1A	Q1B	Q1C	Q1D	Q1E	Q1F	Q2A	Q2B	Q2C	Q2D	Q3A	Q3B	Q3C	Q3D	Q4A	Q4B	Q4C	Q4D	Q4E	1	2	3	4	5
Bairactaris et al (2009)	Y	N	U	N	U	U	Y	Y	N	N	Y	Y	N	N	U	Y	Y	N	U	N	U	Y	Y	Y
Bega et al (2015)	N	Y	Y	N	Y	N	Y	Y	U	N	U	Y	U	N	U	Y	U	Y	U	U	U	Y	Y	N
Bhattacharjee et al (2019)	N	Y	U	N	U	U	U	Y	N	N	Y	U	N	N	U	Y	Y	Y	U	Y	U	Y	Y	N
Crotty et al (2018)	N	U	U	N	U	U	N	Y	N	N	Y	U	N	N	U	U	U	Y	U	N	U	U	Y	N
Garcia Vicente et al (2005)	N	Y	Y	N	U	N	U	Y	N	N	U	U	U	U	U	Y	Y	Y	U	U	U	Y	U	N
Graebner et al (2017)	U	Y	U	N	U	U	N	Y	N	U	U	N	U	U	U	Y	U	Y	U	Y	U	U	U	N
Hesse et al (2006)	N	Y	U	N	U	Y	U	Y	N	N	Y	U	N	N	U	Y	Y	N	U	N	Y	Y	U	Y
Jennings et al (2004)	N	Y	N	N	U	U	Y	Y	N	N	U	Y	U	N	Y	Y	Y	Y	N	Y	U	Y	Y	N
Kupsch et al (2013)	Y	Y	Y	Y	N	N	Y	Y	N	N	Y	Y	N	N	Y	Y	Y	Y	N	Y	U	Y	Y	N
Løkkegaard et al (2002)	Y	Y	Y	N	U	U	Y	Y	N	N	Y	Y	N	N	Y	Y	Y	Y	N	N	U	Y	Y	N
Marek et al (2014)	Y	U	U	N	Y	Y	U	Y	N	N	Y	U	U	Y	U	U	U	Y	U	Y	U	U	U	N
Marshall et al (2006)	N	Y	U	N	U	U	U	Y	N	N	Y	U	U	U	U	Y	U	Y	U	N	Y	Y	U	N
Mirpour et al (2018)	N	Y	U	N	U	U	Y	Y	N	N	U	Y	U	U	U	Y	U	Y	N	U	U	Y	Y	N
Oravivattanakul et al (2016)	U	Y	Y	N	N	N	Y	Y	U	N	U	Y	U	U	U	Y	U	Y	N	Y	U	U	U	N
Sadasivan et al (2015)	Y	Y	Y	N	U	N	Y	U	U	U	U	U	U	U	U	Y	Y	Y	N	U	U	U	U	N
Seifert et al (2013)	U	Y	Y	N	N	N	Y	Y	U	N	U	Y	U	U	U	Y	U	Y	U	U	U	Y	Y	N
Sixel-Doring et al (2011)	Y	Y	Y	N	N	N	U	Y	N	N	Y	U	N	N	U	Y	Y	Y	N	U	U	Y	Y	N
Thiriez et al (2015)	Y	Y	U	N	U	U	U	Y	N	N	U	Y	N	U	U	U	U	Y	U	U	U	Y	Y	N
Tolosa et al (2004)	U	Y	Y	N	N	N	N	Y	N	N	U	Y	U	U	Y	Y	Y	Y	N	Y	U	Y	Y	N
Yomtoob et al (2018)	Y	Y	Y	N	U	U	Y	Y	N	N	U	Y	U	N	U	Y	U	Y	U	N	U	U	Y	N
Green colour = Yes, Red colour = No, Yellow colour = Unclear																								

Supplementary Table 2

Study outcome data for meta-analysis.

STUDY	Population	PD vs. ET	Change in diagnosis	Change in management	PD vs. DIP	Change in diagnosis	Change in management	Early PD	Change in diagnosis	Change in management
Bairactaris et al (2009)	61	NR	NR	NR	NR	NR	NR	NR	NR	NR
Bega et al (2015)	83	18	10	NR	NR	NR	NR	NR	NR	NR
Bhattacharjee et al (2019)	256	18	4	6	8	2	3	190	96	155
Crotty et al (2018)	81	NR	NR	NR	NR	NR	NR	NR	NR	NR
Garcia Vicente et al (2005)	42	NR	NR	NR	NR	NR	NR	NR	NR	NR
Graebner et al (2017)	27	5	2	NR	4	2	NR	NR	NR	NR
Hesse et al (2006)	278	78	60	NR	22	20	NR	129	32	NR
Jennings et al (2004)	35	NR	NR	NR	NR	NR	NR	NR	NR	NR
Kupsch et al (2013)	113	NR	NR	NR	NR	NR	NR	NR	NR	NR
Løkkegaard et al (2002)	58	4	1	NR	4	3	NR	16	1	NR
Marek et al (2014)	701	NR	NR	NR	NR	NR	NR	NR	NR	NR
Marshall et al (2006)	150	NR	NR	NR	NR	NR	NR	NR	NR	NR
Mirpour et al (2018)	134	41	14	NR	15	9	NR	NR	NR	NR
Oravivattanakul et al (2016)	175	14	3	NR	NR	NR	NR	70	30	NR
Sadasivan et al (2015)	65	10	2	2	7	1	4	22	9	18
Siefert et al (2013)	112	NR	NR	NR	NR	NR	NR	NR	NR	NR
Sixel-Doring et al (2011)	125	NR	NR	NR	NR	NR	NR	NR	NR	NR
Thiriez et al (2015)	516	NR	NR	NR	NR	NR	NR	NR	NR	NR
Tolosa et al (2004)	118	NR	NR	NR	NR	NR	NR	NR	NR	NR
Yomtoob et al (2018)	55	NR	NR	NR	51	NR	21	NR	NR	NR

Supplementary Table 3

STUDY	Population	PD vs. vascular	Change diagnosis	Change management	PD vs. LBD	Change diagnosis	Change management	PD vs. dystonia	Change diagnosis	Change management
Bairactaris et al (2009)	61	NR	NR	NR	NR	NR	NR	NR	NR	NR
Bega et al (2015)	83	NR	NR	NR	NR	NR	NR	NR	NR	NR
Bhattacharjee et al (2019)	256	5	2	2	22	12	5	2	0	2
Crotty et al (2018)	81	NR	NR	NR	NR	NR	NR	NR	NR	NR
Garcia Vicente et al (2005)	42	NR	NR	NR	NR	NR	NR	NR	NR	NR
Graebner et al (2017)	27	NR	NR	NR	NR	NR	NR	NR	NR	NR
Hesse et al (2006)	278	15	11	NR	NR	NR	NR	3	1	NR
Jennings et al (2004)	35	NR	NR	NR	NR	NR	NR	NR	NR	NR
Kupsch et al (2013)	113	NR	NR	NR	NR	NR	NR	NR	NR	NR
Løkkegaard et al (2002)	58	NR	NR	NR	NR	NR	NR	6	1	NR
Marek et al (2014)	701	NR	NR	NR	NR	NR	NR	NR	NR	NR
Marshall et al (2006)	150	NR	NR	NR	NR	NR	NR	NR	NR	NR
Mirpour et al (2018)	134	3	2	NR	NR	NR	NR	NR	NR	NR
Oravivattanakul et al (2016)	175	NR	NR	NR	NR	NR	NR	NR	NR	NR
Sadasivan et al (2015)	65	5	1	4	NR	NR	NR	NR	NR	NR
Siefert et al (2013)	112	NR	NR	NR	NR	NR	NR	NR	NR	NR
Sixel-Doring et al (2011)	125	NR	NR	NR	NR	NR	NR	NR	NR	NR
Thiriez et al (2015)	516	NR	NR	NR	NR	NR	NR	NR	NR	NR
Tolosa et al (2004)	118	NR	NR	NR	NR	NR	NR	NR	NR	NR
Yomtoob et al (2018)	55	NR	NR	NR	NR	NR	NR	NR	NR	NR

Supplementary Table 4

Agreement between DaTscan and clinical assessment (only studies that reported results are shown).

STUDY	Population (n=)	Agreement between DaTscan and clinical (kappa)	Agreement between DaTscan and clinical
Bairactaris et al (2009)	61	0.229	65%
Jennings et al (2004)	35	NR	74%
Oravivattanakul et al (2016)	175	NR	57%
Sixel-Doring et al (2011)	125	NR	87%
Yomtoob et al (2018)	55	NR	85%

Supplementary Table 5

Relationship between time since onset of symptoms and change in management (in blue) and change in diagnosis (orange).

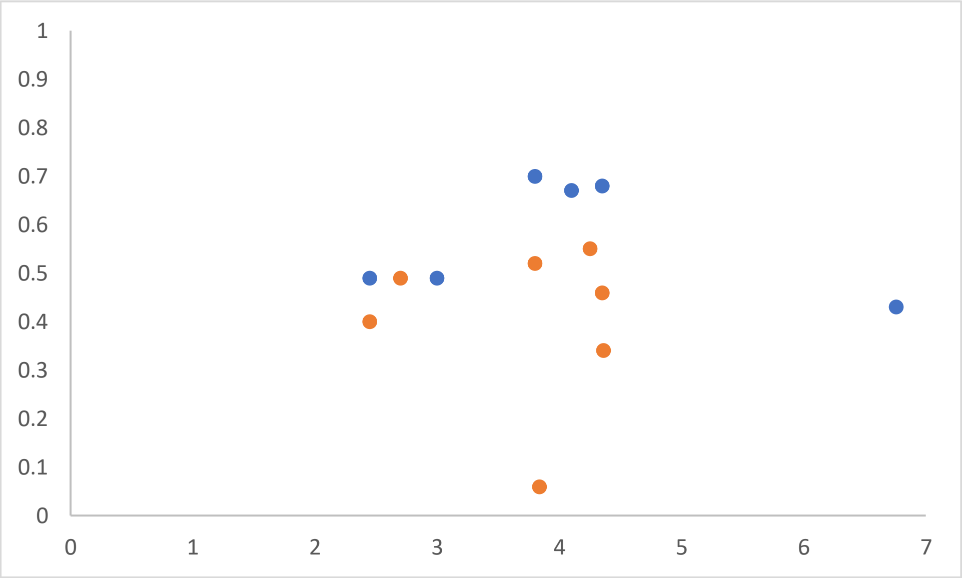

Supplementary Figure 2

Subgroup analyses forest plots

Region: North America vs. Europe

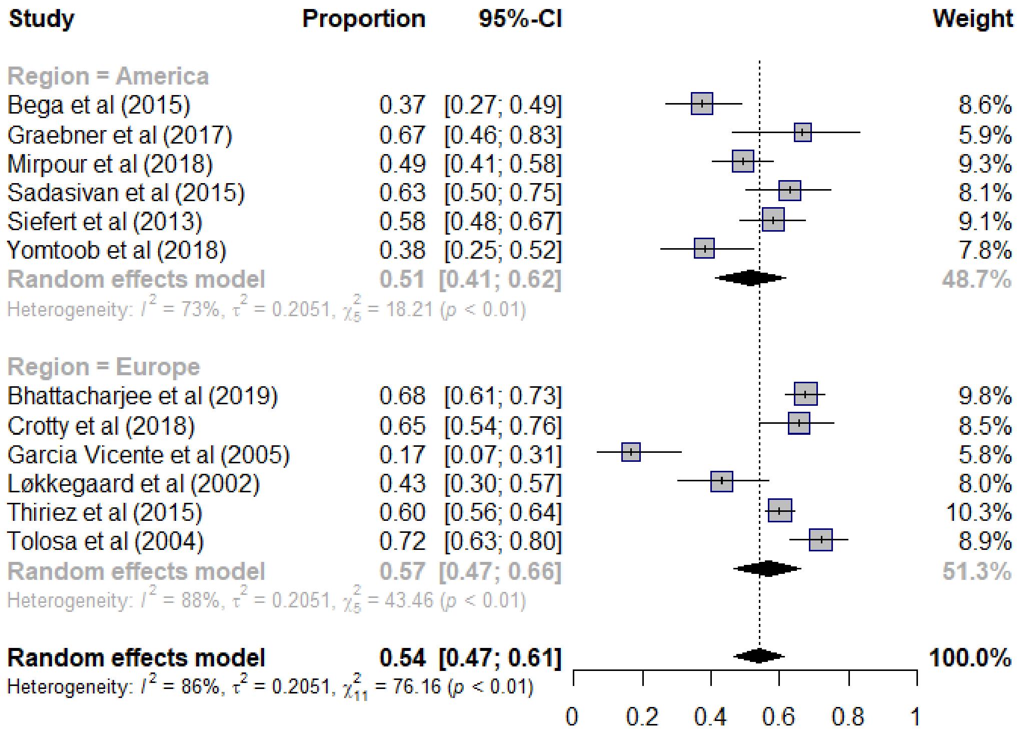

Supplementary Figure 3

Study design: Retrospective, single-arm vs. other

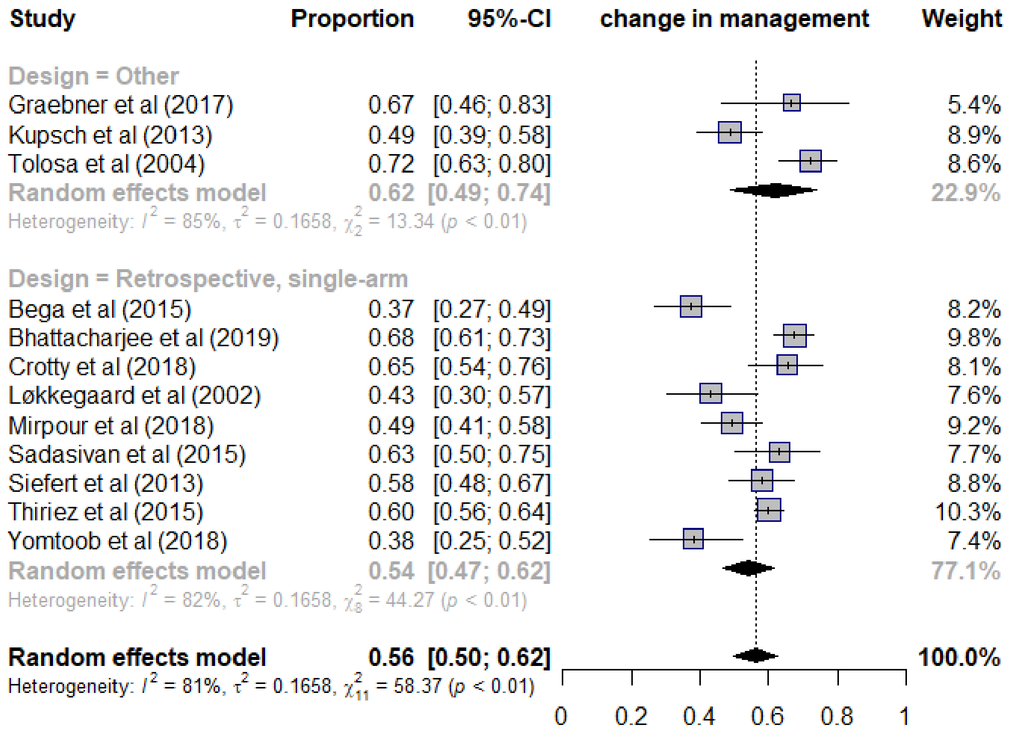

Supplementary Figure 4

Mean age of patients: < 64 years vs. ≥ 64 years

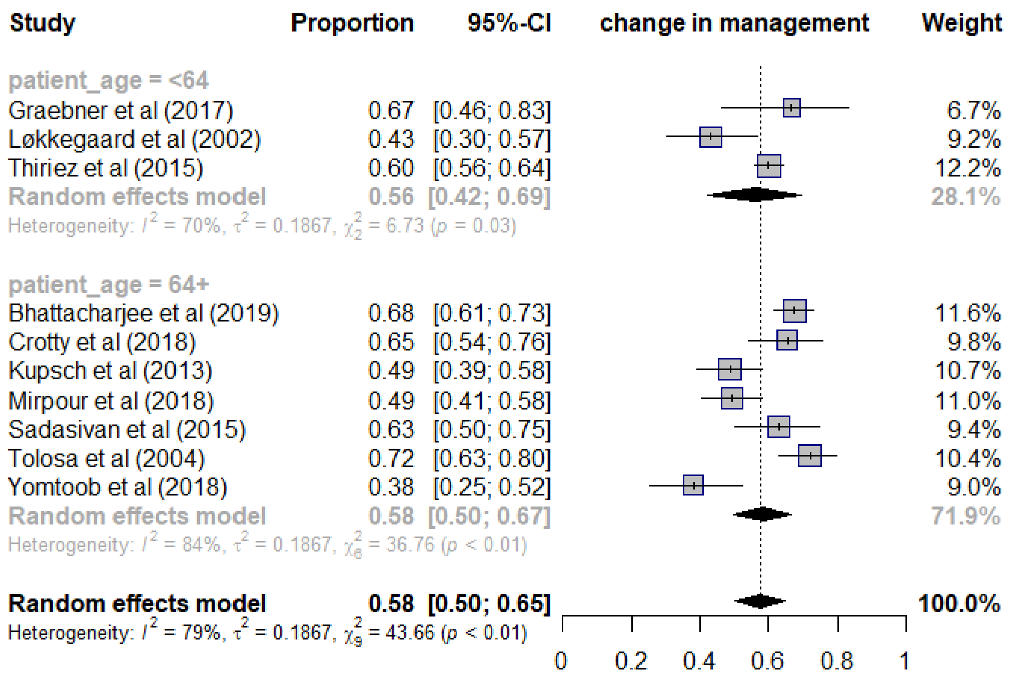

Supplementary Figure 5

Time since onset of first symptoms: >3.84years vs. equal or ≥3,84years

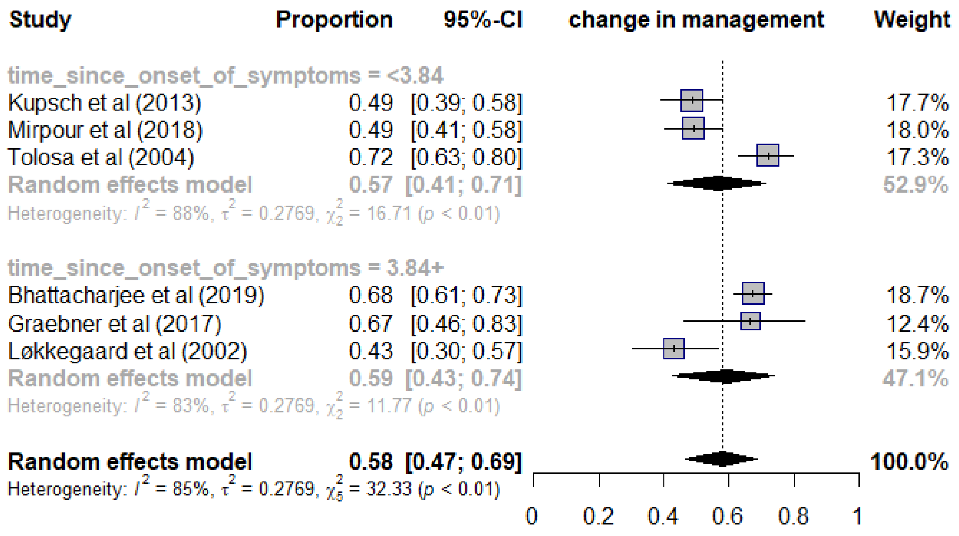

Supplementary Figure 6

Female to male ratio: <1 vs. ≥ 1

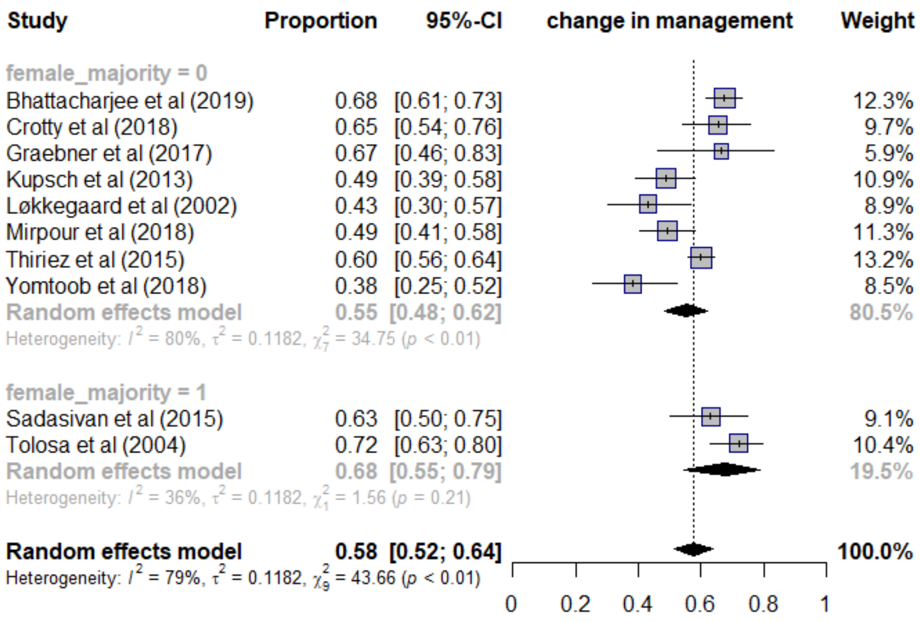

Supplementary Figure 7

Follow-up time: <16 months vs. ≥ 16 months

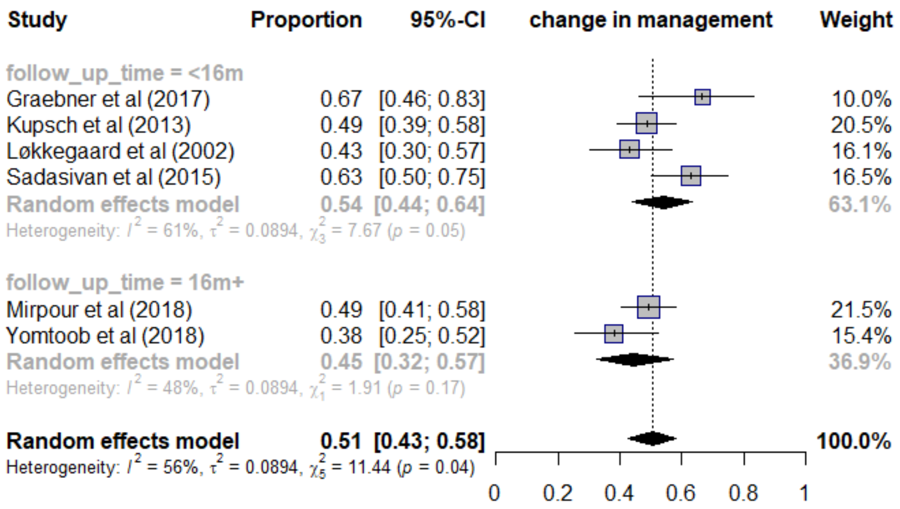

Supplementary Figure 8

PD vs ET

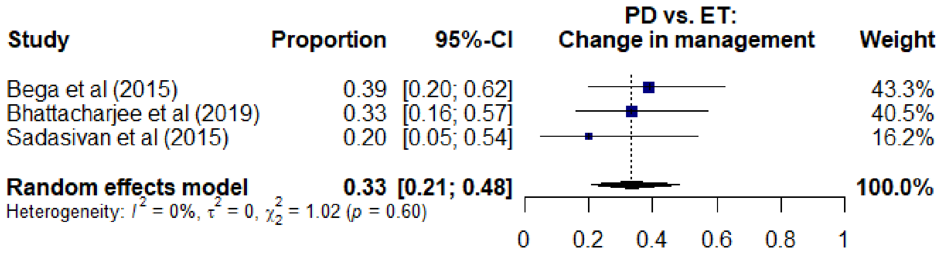

Supplementary Figure 9

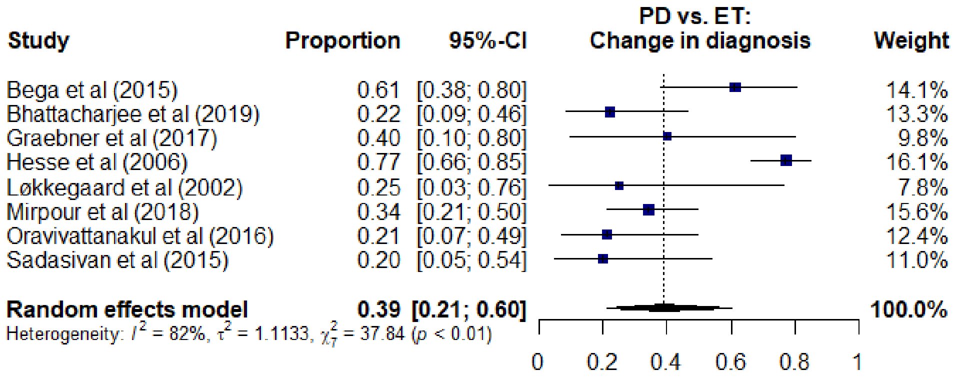

Supplementary Figure 10

PD vs DIP

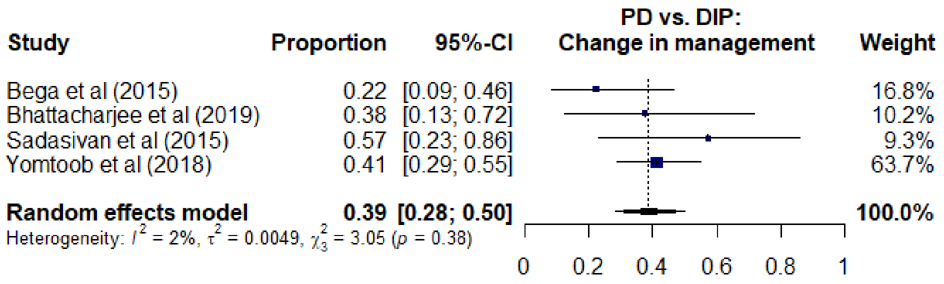

Supplementary Figure 11

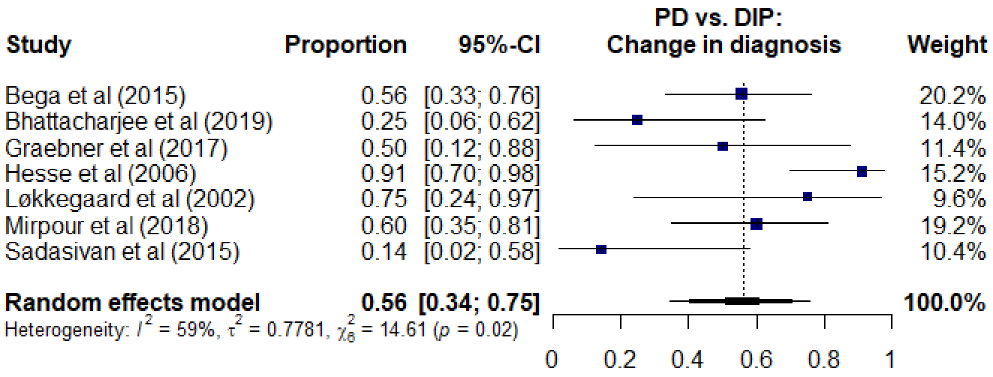

Supplementary Figure 12

PD vs vascular

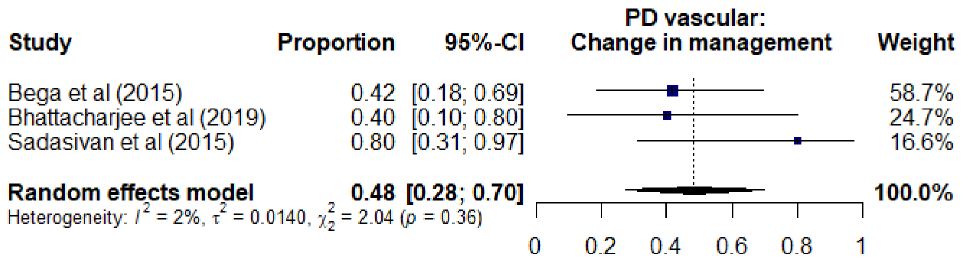

Supplementary Figure 13

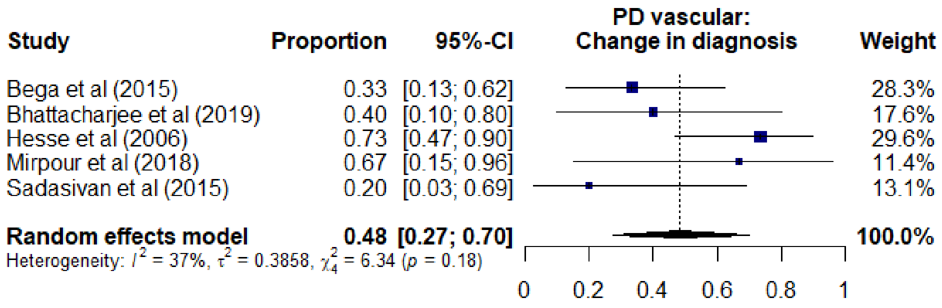

Supplementary Figure 14

PD vs Early

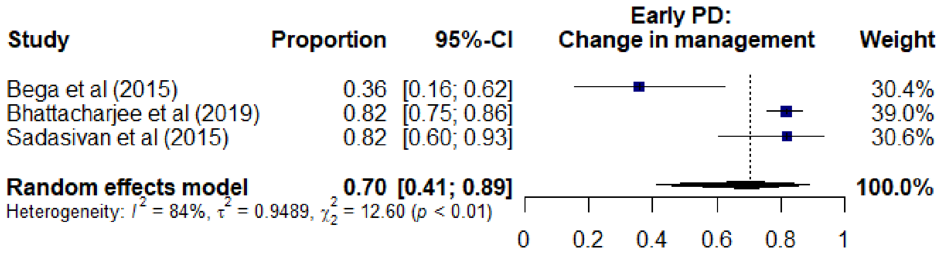

Supplementary Figure 15

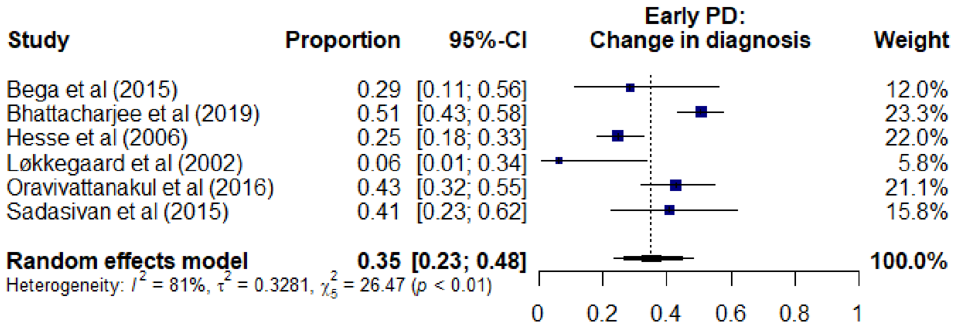

Supplementary Figure 16

Leave-one-out sensitivity analyses

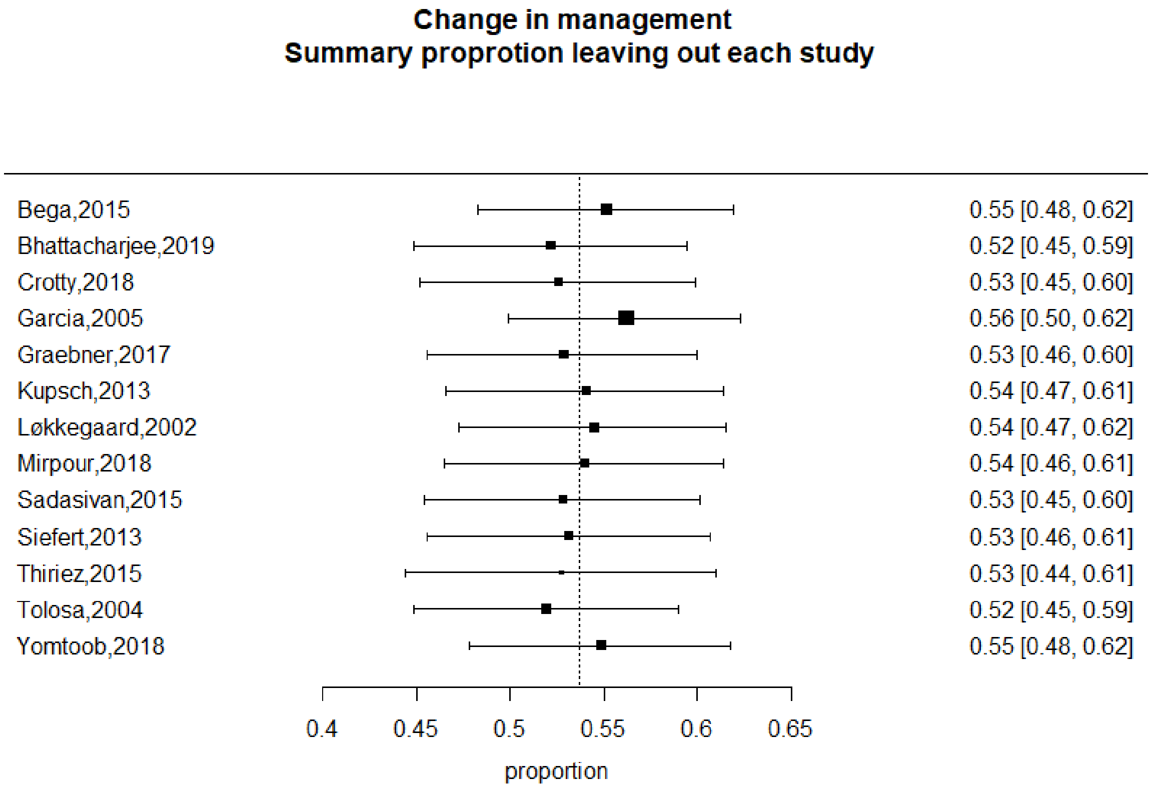

Supplementary Figure 17: Summary of leave-one-out sensitivity analysis for change in management.

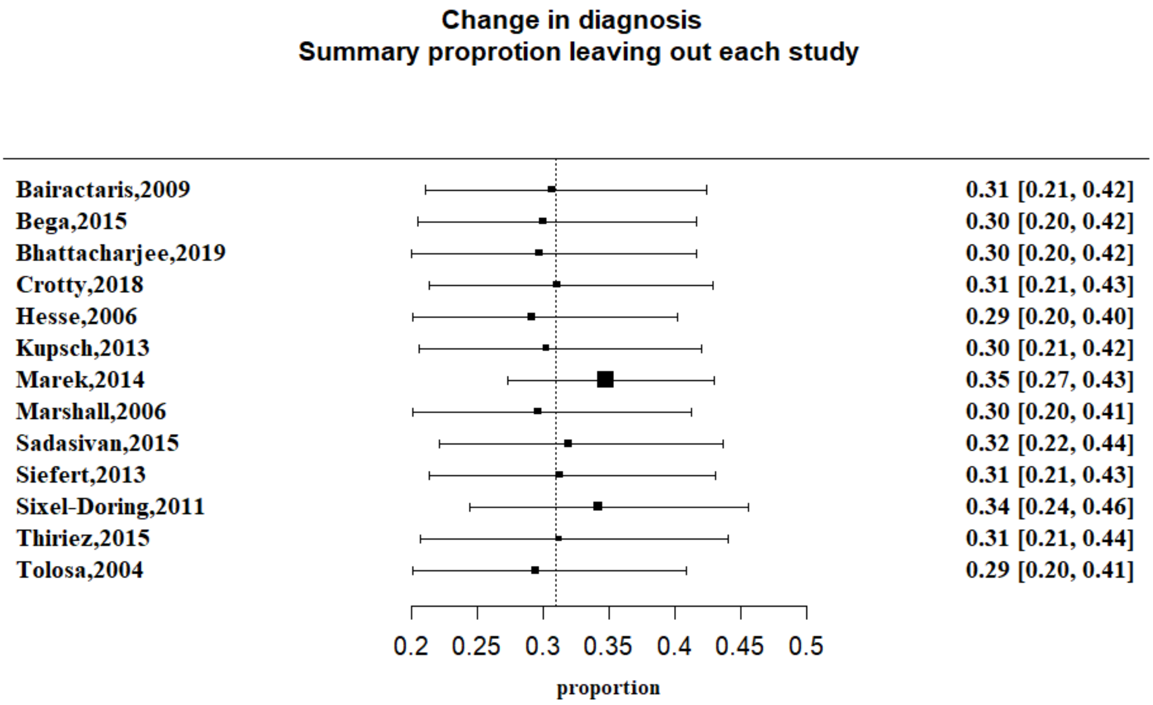

Supplementary Figure 18: Summary of leave-one-out sensitivity analysis for change in diagnosis.

Univariable meta-regression results

Change in management

Female to male ratio

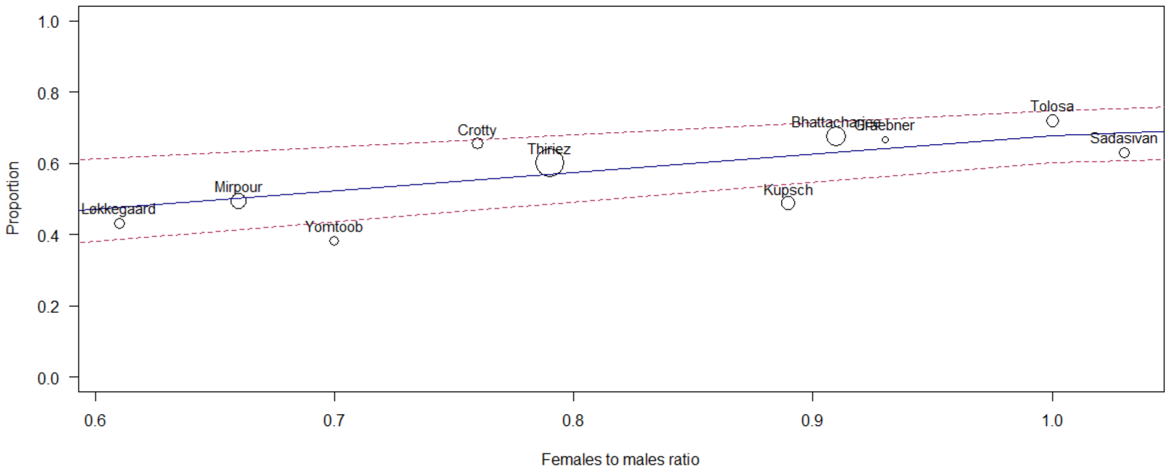

Supplementary Figure 19

	Point estimate B	p-value	95%CI Lower bound	95%CI Upper bound
Intercept	-1.645	0.011	-2.927	-0.364
Predictor	2.387	0.002	0.856	3.920

Supplementary Table 6: Meta-regression with random effects model – coefficients. Outcome variable logit transformed proportions

Change in diagnosis

Mean age of patients

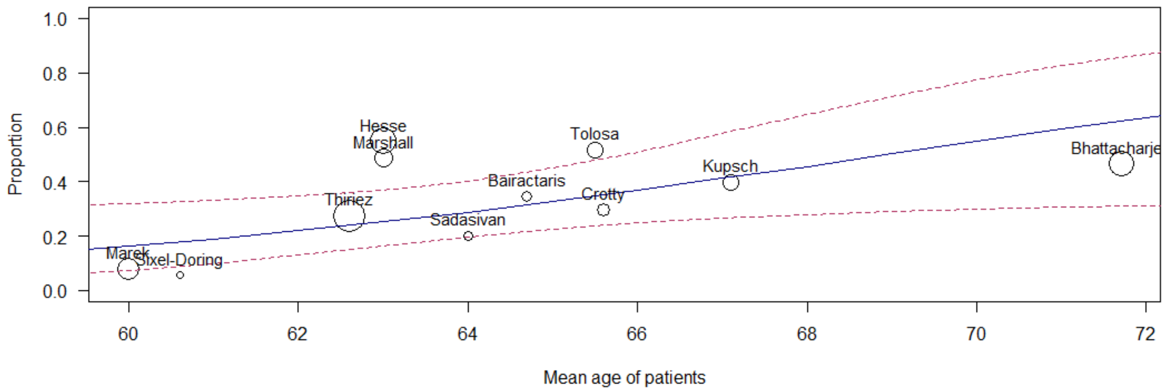

Supplementary Figure 20

	Point estimate B	p-value	95%CI Lower bound	95%CI Upper bound
Intercept	-12.589	0.0191	-23.118	-2.0589
Predictor	0.183	0.0285	0.0192	0.3459

Supplementary Table 7: Meta-regression with random effects model – coefficients. Outcome variable logit transformed proportions

Female to male ratio

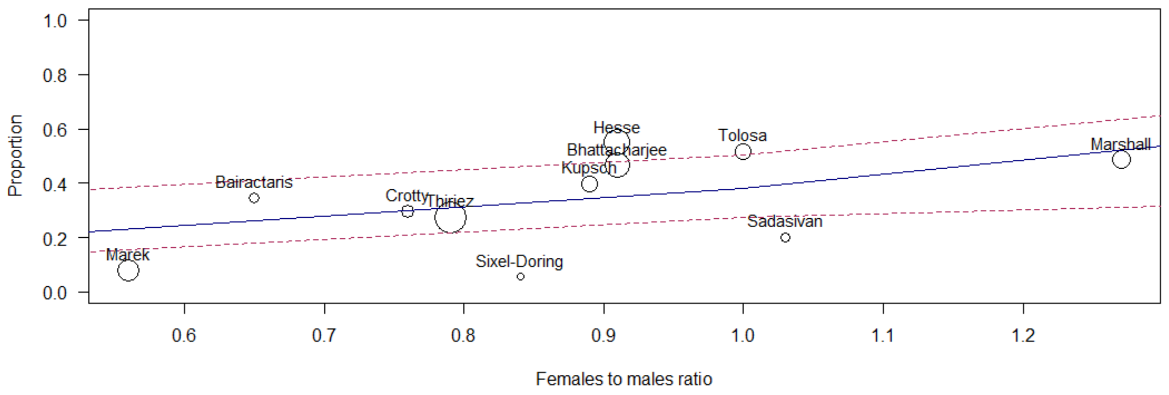

Supplementary Figure 21

	Point estimate B	p-value	95%CI Lower bound	95%CI Upper bound
Intercept	-3.1473	0.0018	-5.1262	-1.1685
Predictor	2.6615	0.0185	0.4471	4.8759

Supplementary Table 8: Meta-regression with random effects model – coefficients. Outcome variable logit transformed proportions

Predictor	Change in management (p-value)	Change in diagnosis (p-value)
Year of publication	0.285	0.266
Sample size	0.224	0.102
Study region	0.487	0.152
Study design	0.308	0.837
Mean age	0.229	0.028
Female to male ratio	0.002	0.018
Time of follow-up	0.070	0.931
Clinical assessment	0.788	0.533
Image interpretation	0.190	0.0002
Time since onset of symptoms	0.784	0.874

Supplementary Table 9

Publication bias: funnel plot

Logits

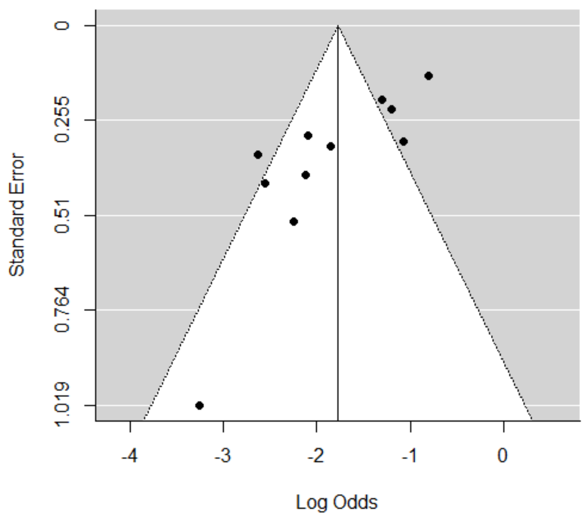

Proportions

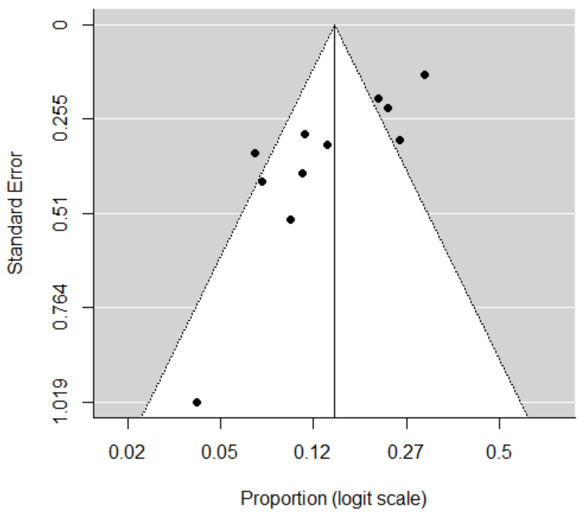

Supplementary Figure 22

Mixed-effects meta-regression model	z-score	P-value
predictor: standard error	-4.117	< 0.0001

Supplementary Table 10: Regression Test for Funnel Plot Asymmetry

References

Borenstein, M., Hedges, L. V., Higgins, J., & Rothstein, H. R. (2009). *Introduction to Meta-Analysis*. West Sussex, United Kingdom: John Wiley & Sons, Inc.

DerSimonian, R., & Laird, N. (1986). Meta-analysis in clinical trials. *Controlled Clinical Trials*, *7*(3), 177–188.

Hardy, R. J., & Thompson, S. J. (1996). A likelihood approach to meta-analysis with random effects. *Statistics in Medicine*, *15*(6), 619–629.

Higgins, J., & Thompson, S. (2002). Quantifying heterogeneity in a meta-analysis. *Statistics in Medicine*, *21*(11), 1539–1558.

Raudenbush, S. W., & Bryk, A. S. (1985). Empirical Bayes Meta-Analysis. *Journal of Educational Statistics*, *10*(2), 75–98.

Raudenbush, S. W., & Bryk, A. S. (2002). *Hierarchical Linear Models: Applications and data analysis methods* (2nd editio). Thousand Oaks, California: Sage Publications, Inc.

Schwarzer, G. (2010). meta: General Package for Meta-Analysis. Retrieved from https://cran.r-project.org/web/packages/meta/index.html

Schwarzer, G., Carpenter, J. R., & Rücker, G. (2015). *Meta-Analysis with R*. Cham Heidelberg New York Dordrecht London: Springer.

Viechtbauer, W. (2010). Conducting Meta-Analyses in R with the metafor Package. *Journal of Statistical Software*, *36*(3), 1–48.

Wang, N. (2018). How to Conduct a Meta-Analysis of Proportions in R: A Comprehensive Tutorial. https://doi.org/10.13140/RG.2.2.27199.00161
